# Food addiction, orthorexia nervosa and dietary diversity among Bangladeshi university students: a large online survey during the COVID-19 pandemic

**DOI:** 10.1186/s40337-022-00680-0

**Published:** 2022-11-14

**Authors:** Mst. Sadia Sultana, Md. Saiful Islam, Abu Sayeed, Kamrun Nahar Koly, Katya Baker, Rakib Hossain, Sanjida Ahmed, Most. Zannatul Ferdous, Mahfuza Mubarak, Marc N. Potenza, Md Tajuddin Sikder

**Affiliations:** 1 Department of Public Health and Informatics, Jahangirnagar University, Savar, Dhaka 1342 Bangladesh; 2 Center for Advanced Research Excellence in Public Health, Savar, Dhaka 1342 Bangladesh; 3 Department of Post-Harvest Technology and Marketing, Patuakhali Science and Technology University, Patuakhali, 8602 Bangladesh; 4 Health System and Population Studies Division, International Centre for Diarrhoeal Disease Research, Bangladesh (icddr,b), Mohakhali, Dhaka, 1212 Bangladesh; 5 King’s College London, London, UK; 6 Department of Psychiatry and Child Study Center, Yale School of Medicine, New Haven, CT USA; 7 Connecticut Mental Health Center, New Haven, CT USA; 8 Connecticut Council on Problem Gambling, Wethersfield, CT USA; 9 Department of Neuroscience and Wu Tsai Institute, Yale University, New Haven, CT USA

**Keywords:** Food addiction, Orthorexia nervosa, Dietary diversity, Students, Bangladesh, COVID-19, Addictive behaviors

## Abstract

**Purpose:**

Maladaptive eating behaviors like food addiction (FA) are common among students, and orthorexia nervosa (ON) is being evaluated as a new condition among eating disorders (EDs). Moreover, dietary diversity (DD) is recognized as an important component of a healthy diet. Thus, the current study sought to examine the prevalence of ON and FA as well as the factors associated with ON, FA, and DD among university students in Bangladesh during the COVID-19 pandemic.

**Methods:**

Four-thousand-and-seventy-six students were recruited and completed an online survey consisting of the Bratman Orthorexia test, the modified Yale Food Addiction Scale, and a questionnaire based on the Food and Agriculture Organizations’ guidelines. Inferential statistics, bivariate and multivariable linear regression were used for analyzing data.

**Results:**

ON and FA were observed in 1.7% and 7.5% of participants, respectively, with 43.8% exhibiting Health Fanatic eating attitude. The mean DD score was 5.96 (SD = 1.56). Students with ON consumed more legumes, nuts, seeds, and vegetables including dark green leafy vegetables whereas students with FA consumed more organ meats and eggs. Students who were older-aged, married, formerly smoked, had fitness goals, and had guilty feelings about violating food rules were more likely to have ON, whereas those who were female, were married, actively smoked, and were overweight and obese were more likely to have FA.

**Conclusions:**

The findings suggest that students from Bangladesh are at risk of FA, and less so for ON. These entities and low DD deserve more research attention in Bangladesh to increase awareness and ensure appropriate interventions.

## Plain English summary

Eating disorders, maladaptive eating behaviors, and low diversified diets are major public health issues amid the COVID-19 pandemic among university students. The current study aimed to address these issues by investigating Orthorexia Nervosa (ON), food addiction (FA), and dietary diversity. Four-thousand-and-seventy-six students completed an online questionnaire. It was found that being underweight was associated with ON. On the other hand, being overweight/obese was associated with having FA. Individuals who were former smokers and who had poor social interaction were significantly associated with having Orthorexia Nervosa. 1st-year university students are more likely to have FA compared to 5th-year/master’s students. Active smokers were more likely to be food addicted. Students who had FA had less guilty feelings about violating food rules. Participants who had FA consumed more organ meats and eggs. Students who were from upper socio-economic status were more likely to have higher dietary diversity. Nutritional awareness programs focusing on DD and healthy eating habits should be implemented for students.

## Background

Eating disorders (ED) [1] have been understudied in the global health sector, despite being debilitating conditions with significant consequences, including increased mortality, reduced social functioning, and employment problems [2, 3]. EDs are associated with long-term impairments, including psychosocial illnesses such as depression and anxiety [4], with substantially increasing prevalence rates among young adults [5]. It is estimated that 10%-20.6% of young adults in South-East Asian nations are at risk of developing an ED such as anorexia [6]. In addition to anorexia, orthorexia Nervosa (ON) is a new form of disordered eating behavior, and younger adults are more susceptible to ON [7]. ON is characterized by a pathological fixation with ‘proper’ nutrition, which can lead to inadequate diet and significant medical issues [8]. Although ON has been recognized as a distinct disorder [9], many features of ON echo symptoms of anorexia and obsessive–compulsive disorder (OCD) stimulating debate if orthorexia is a distinct disorder or a subset anorexia or OCD [9]. Goal-oriented attitude, viewing diet adherence as a sign of self-discipline and deviation from the diet as a lack of self-control are some common symptoms that both anorexia and orthorexia share [10]. In terms of overlap with OCD, people with higher score on ON measures exhibit obsessive–compulsive symptoms, obsessive tendencies: recurring, bothersome thoughts concerning food and health at unexpected times, exaggerated anxiety about pollution and impurity, as well as a pressing need to organize meal and eat it in a ritualized way [10, 11]. However, there are significant differences between orthorexia, as well as many additional disorders. Unlike most individuals with anorexia nervosa or bulimia nervosa, who are preoccupied with the amount of food they eat as well as their physical appearance, people who score highly on measures of orthorexia are more worried about the quality of their food than the quantity [12]. Furthermore, people who score highly on measures of orthorexia spend a significant amount of time (more than three hours per day) on food research, analysis, and preparation, depending on the individuals’ adherence to preconceived notions of ‘health’ [10]. In severe circumstances, people who score highly on measures of orthorexia would rather starve than consume food they deem wrong or unhealthy [13], which can result in unintended malnutrition [14]. Other significant detrimental impacts of ON include decreased quality of life, social alienation, and dissatisfaction in relationships [8]. However, there are no comprehensive or standardized criteria for diagnosing ON [15], and the DSM-5 expanded did not include ON [16], making its diagnosis and treatment more difficult. Bratman’s Orthorexia Test (BOT) can be used to measure ON [12] where the average BOT scores of subjects falling near the cut-off point for a Health Fanatic eating attitude can suggest orthorexic tendencies [17]. Those who exhibit Health Fanatic eating attitude may have a risk of ON [18]. Previous studies have found that 7.8% students from China had ON [19], 81% students from rural areas in US had orthorexic tendencies [20], and 26.6% dietetics students had food fanaticism (proneness to developing ON) [21]. In addition, obesity and overweight [22], increased exercise frequency, younger age, vegetarian diet, and consuming a specialized diet [20] have been associated with ON.

Different EDs have been associated with different behaviors. For example, individuals with binge eating disorder (BED) may overestimate their body size and display compulsive eating in absence of any compensatory weight-reducing behaviors (e.g., purging) [23]. BED overlaps with food addiction (FA), with 56.8% of participants with BED and obesity also having FA in one study [24]; thus, there is a need to consider FA in the disordered eating spectrum [25]. FA has aroused both scientific and public policy attention, due to its relationship to obesity and population weight management [26]. Although both ON and FA are not formally recognized as a psychiatric diagnosis yet but it can cause many negative health consequences [9, 27]. For example, individuals who score highly on a measure of FA typically have problems limiting consumption of highly palatable processed foods, leading to overeating and obesity [28] and negative health effects including diabetes mellitus, hypertension, and heart disease [29]. A recent systematic review reported the prevalence of FA in young adults (under 35 years old) as ranging from 7.8% to 25% [30]. FA has been found to be higher among individuals who are female [31], single [32], actively smoking [33], obese and overweight [34]. This addictive-like food consumption pattern is a major concern for young adults, particularly in emerging nations like Bangladesh where fast food has become increasingly popular [35].

Low levels of dietary diversity (DD) present a similar public health challenge for young adults in Bangladesh [36]. Poor dietary behaviors [37], parental control, and food awareness, and the burden of academic, professional, and social life [38] may make younger adults particularly vulnerable to low DD. In Bangladesh, cereals (mostly rice) are meal staples, accounting for almost two-thirds of the daily diet. Typically the diet also includes small portions of vegetables, a small quantity of pulses (legumes), and very little protein in the forms of milk, milk products, and meat [39]. As a consequence, traditional eating patterns of Bangladeshi young adults seldom translate into healthy, balanced diets [40]. A prior Bangladeshi study during the COVID-19 pandemic showed that rural participants, with low average educational attainments and monthly incomes, had lower DD than the participants with lower average educational attainments and monthly incomes [41]. Thus, increasing DD may be important for improving micronutrient nutrition among this population in Bangladesh [39]. Moreover, ensuring healthy diets can help students achieve optimal educational attainments [42].

A well-balanced and diverse diet is also important for ensuring healthy immune systems, particularly relevant during the COVID-19 pandemic [43]. The COVID-19 pandemic has triggered dysfunctional dietary behaviors in many forms and particularly affected people who were already diagnosed with EDs [44]. Alcohol and cigarette consumption increased in some populations, particularly during quarantines or lockdowns [45]. Some people have consumed extra meals and high-calorie foods to deal with pandemic-related anxiety, potentially generating or worsening FA [46, 47]. In addition to FA, new difficulties for families and individuals may have emerged during the COVID-19 pandemic, including poverty and economic turmoil which may promote reduced DD, and this which is particularly pertinent to lower-and-middle income countries like Bangladesh [41, 48].

Although studies have examined DD in adolescents, students and the general population of Bangladesh [41, 49, 50], only one study has explored DD during the COVID-19 pandemic [41]. Furthermore, there is only one study on FA in the general population [51], but no studies that explore FA among students in Bangladesh. Although the high prevalence of EDs continues in high-income western nations, a global trend has been noted in densely populated Asian regions [52]. Frequent occurrence of disordered eating behaviors, including ON, in Bangladesh may be associated with economic changes, globalization, increased access to fast food and greater exposure to western cultures [35, 53, 54]. Despite having a distinct behavioral pattern, ON has not been investigated in Bangladesh. It is important to understand the prevalence and correlates of ON so that it can be successfully managed and resources can be allocated for awareness, education, prevention and treatment. Overall, there is limited information regarding ON, FA and DD amid the COVID-19 pandemic, and an investigation into these issues is needed in Bangladesh. Therefore, this study aimed to explore the prevalence of ON and FA as well as the factors associated with ON, FA and DD in Bangladesh during the pandemic. The findings of this study should aid government agencies in the development and implementation of policies and practices to mitigate the negative consequences of ON, FA, and low DD.

## Methodology

### Study design and participants

This study used an online cross-sectional survey design and convenience sampling technique. Participants were university students, and the inclusion criteria were: (1) being 18 years old or older, (2) having ability to read Bengali language, (3) having internet access, and (4) residing in Bangladesh during the survey period. Incomplete surveys and being under 18 years were exclusion criteria.

### Sampling

Utilizing a single sample proportion test, the sample size was calculated. Based on the intended level of significance (Z), a margin of error (d), and the expected level of the proportion (p), the test estimates the minimal sample size (n) required to determine a proportion in a source population. The following assumptions were considered during the calculation: (1) 50% predicted prevalence among Bangladeshi university students was used (*p* = 50%) due to the lack of previous investigations on FA, ON and DD in the country. (2) 95% confidence level (Z = 1.96) and (3) 5% margin of error (d = 0.05). The formula is as follows:

$${\text{n}} = \frac{{{\text{z}}^{2} \times {\text{p}} \times (1 - {\text{p}})}}{{{\text{d}}^{2} }} = \frac{{(1.96)^{2} \times 0.5 \times (1 - 0.5)}}{{(0.05)^{2} }} = \, 384.16 \approx 384.$$

By taking a 10% non-response rate for possible compensation of non-responses, an optimal sample size of 422 was obtained. We sought to gather more samples than our estimated sample size to strengthen the external validity and generalizability of the study [55]. However, a total of 4,286 university students across Bangladesh participated in the survey, and after eliminating incomplete or ineligible data, data from 4076 respondents were included in the final analyses.

### Procedure

The survey was conducted between January and February 2021. A presurvey orientation training session focused on survey methods, and data collection methods were delivered by lead members of the research team for 47 research assistants from different Bangladeshi universities. The study protocol was approved by the Biosafety, Biosecurity & Ethical Committee, Jahangirnagar University, Dhaka, Bangladesh [Ref No: BBEC, JU/M 2021//3(2)]. The study followed the Checklist for Reporting Results of Internet ESurveys (CHERRIES) guidelines [56]. An e-questionnaire was employed as the data collection tool. The questionnaire was developed in English initially, then translated into the local language (Bengali) by a bilingual member of the research team, which was double-checked by another bilingual team member. A third independent bilingual translator back-translated the material. Pre-testing was done among 40 participants before starting the final data collection to ensure consistency and avoid/limit biases. After the finalization of the questionnaire, the research assistants circulated the e-questionnaire link among their academic networks using social media (e.g., Facebook, What’s app, Instagram, Viber, Telegram, etc.). To comply with COVID-19 movement restriction during the date collection period, data were collected using convenience sampling technique. Participants were automatically directed to the cover page and an informed consent page after clicking on the link. The cover pages of the questionnaires briefly explained the study and gave instructions regarding how to complete the questionnaire. The cover page stated that only university students could participate in the survey. The cover page also specified that data from the survey would be used only for research purposes and that participation was entirely voluntary. The anonymity and confidentiality were maintained, and data privacy was ensured through storage of data in a password-protected folder.

### Measures

The questionnaire was divided into five sections with questions and instruments assessing: (1) socio-demographic information, (2) lifestyle and associated domains, (3) DD based on guidelines proposed by the Food and Agriculture Organization [57], iv) ON, and v) FA.

#### Socio-demographic information

The first part of the questionnaire collected sociodemographic data, including age, gender, academic year of study, relationship status (single/married/in a relationship), subject area (science/arts/commerce), socio-economic status (SES, categorized based on monthly family income: lower SES < 15,000 Bangladeshi Taka [BDT], middle SES = 15,000–30,000 BDT, and upper SES > 30,000 BDT) [58], and residence (urban/rural). Self-reported measures of height and weight [59] were recorded. Body mass index (BMI) was determined as weight (kg) divided by the square of height in meters and classified as underweight (BMI < 18.5 kg/m^2^), normal weight (BMI = 18.5–24.9 kg/m^2^), overweight (BMI = 25.0–29.9 kg/m^2^), and obese (BMI > 30 kg/m^2^) [60–62].

#### Lifestyle-related variables

Lifestyle-related variables were obtained from questions about having fitness goals (yes/no), tobacco-smoking behaviors (active/former/non), and perceived social interaction (good/moderate/poor). Participants were asked if they had guilty feelings regarding food rules violations with three response options: always, sometimes, and no. Food rules depend upon individual adherence to preconceived “healthy” notions of food and diet (i.e., eating processed foods, consuming high quantity of fat, sugar and salts) [8]. Moreover, participants were asked to report their perceived weight during the survey period compared to the pre-COVID-19 period (gain/loss/same/do not know). Perceived problem-solving skill was also asked from the participants with a close-ended question including three response options (good/moderate/poor).

#### The Bratman Orthorexia test (BOT)

The Bratman Orthorexia test (BOT) [10] was used to measure ON. It consists of ten dichotomous items with “yes/no” responses (e.g., *"I feel guilty if I deviate from my dietary habits”*). Each “yes” response corresponds to one point; participants with fewer than 5 points were considered healthy, those with 5–9 points were considered as having Health Fanatic eating attitude to eating, and those with 10 points were considered as having ON [59]. This scale has previously been used among students [59]. The internal consistency of the scale was previously found to be adequate [63]. The Cronbach alpha of this tool was 0.71 for the present study.

#### The modified Yale Food Addiction Scale (mYFAS)

The modified Yale Food Addiction Scale (mYFAS), a short form of the Yale Food Addiction Scale (YFAS) [64], was used to measure FA. The scale consists of nine items, including seven items to assess criteria of substance dependence (adopted for food) as defined by the DSM-IV (control, attempts, time, activities, problems, tolerance, withdraw, and impairment), and two other items to determine the existence of clinically significant impairment or distress [65]. Symptoms were evaluated with respect to the previous 12 months. Someone may be considered to have FA when when at least three of the seven symptoms and a clinically significant impairment or distress is present [65]. It has been evidenced that the psychometric characteristics of the mYFAS are comparable to the original YFAS [64]. For the mYFAS scale, the sensitivity (92.3%) and negative predictive value (99.5%) for diagnosing FA have been reported as excellent [66]. The Cronbach alpha of this tool was 0.74 for the present study.

#### Dietary diversity (DD)

The dietary diversity (DD) score was calculated using the FAO (2013) guidelines, which advocates using a questionnaire to assess DD [67]. The questionnaire focuses on nine major food groups: (1) cereals, (2) dark green, leafy vegetables, (3) vitamin A-rich fruits and vegetables, (4) other fruits and vegetables, (5) organ meat, (6) meat, fish, and seafood, (7) eggs, (8) legumes, nuts, and seeds, and (9) milk and milk products, with a focus on the past 24 h. DD was estimated based on a half-serving of at least one item from each of the food groups in the past 24 h. This tool has been previously used in a developing country, Iran [68]. DD was calculated as the sum of all food groups. Each food group consumed scored one, with a maximum score of nine [68]. The Cronbach alpha of this tool was 0.77 for the present study.

### Statistical analysis

The data were analyzed using IBM-SPSS for Windows, version 25 (Chicago, IL, USA). Descriptive statistics including frequencies, percentages, means, and standard deviations [31] were used to show the sample characteristics of the study population. A t-test was employed to measure significant group differences relating to FA and ON with respect to consumption of different food groups. Bivariate and multivariable linear regression analysis was performed considering FA, ON, and DD as the dependent variables. All the assumptions of linear regression (linearity, homoscedasticity, and multicollinearity) were checked. The strengths of the estimated associations were estimated by Beta co-efficient with 95% confidence interval (CI) and *p*-valued. Statistical significance was evaluated as *p* ≤ 0.05 for all tests in this exploratory study.

## Results

### General profile of participants

Respondents included 4,076 participants aged between 18 and 28 years with a mean age of 22.07 (SD = 1.69). Of these, 54.9% were female and most (77.3%) were single. Many participants were 2nd year students (28.0%), came from science backgrounds (68.7%), belonged to upper SES (50.0%), and resided in urban areas (78.7%). With regard to BMI, participants were classified as underweight (12.4%), normal/lean (64.9%), overweight (15.1%) and obese (7.6%).

A sizeable majority had specific fitness goals (72.2%), and 42.9% reported that they had gained weight compared to pre-COVID-19 period. Many reported having guilty feelings about violating food rules (38.3%), moderate problem-solving skills (59.7%), and good social interactions (45.5%). With regard to tobacco-smoking, most (84.8%) did not smoke, with minorities reporting active (11.0%) and former (4.2%) smoking.

### Orthorexia nervosa

ON were observed in 1.7% of participants, with 43.8% exhibiting Health Fanatic eating attitude. The mean score of the Bratman Test for Orthorexia (BOT) scale was 4.41 (SD = 2.15) out of 10. All studied variables (with *p* < 0.25 in bivariate regression analyses) were considered in multivariable regression analyses (Table 1). Based on multivariable regression analyses, orthorexia was significantly associated with participants who were older aged (24–28 years), in their 1^st^ or 2^nd^ year of university, married, had formerly smoked, were of lower SES, had normal weight (compared to underweight), had fitness goals, had lost weight compared to the pre-COVID-19 period, experienced feelings of guilt when violating food rules, and had good problem-solving skills and social interactions (Table 1). Participants who scored highly on a measure of orthorexia consumed more legumes, nuts and seeds (t = 29.10, *p* < 0.001), dark green leafy vegetables (t = 45.19, *p* < 0.001), vitamin A rich fruits and vegetables (t = 62.72, *p* < 0.001), milk and milk products (t = 17.39, *p* < 0.001) (Table 2).

**Table 1:** Descriptive analyses of all studied variables and regression analyses (bivariate and multivariable) by orthorexia

Variables	Overall N = 4076	Orthorexia										
		Mean (31)	Unadjusted estimates					Adjusted estimates^‡^				
	n (%)		B	SE	t	ꞵ	*p*-value	B	SE	t	ꞵ	*p*-value
*Age*												
18–20 years	727 (17.8)	4.34 (2.22)				†						†
21–23 years	2553 (62.6)	4.33 (2.12)	− .01	.09	− 0.13	< − .01	.898	.07	.10	0.70	.02	.487
24–28 years	796 (19.5)	4.7 (2.13)	.36	.11	3.28	.07	**.001**	.43	.14	3.11	.08	**.002**
*Sex*												
Male	1840 (45.1)	4.36 (2.14)				†						†
Female	2236 (54.9)	4.45 (2.15)	.09	.07	1.31	.02	.189	.10	.07	1.36	.02	.173
*Academic year of study*												
5th year/ Master	395 (9.7)	4.65 (2.04)				†						†
1st year	682 (16.7)	4.48 (2.15)	− .17	.14	− 1.21	− .03	.224	.40	.16	2.54	.07	**.011**
2nd year	1143 (28.0)	4.47 (2.19)	− .17	.13	− 1.38	− .04	.166	.30	.14	2.14	.06	**.032**
3rd year	1066 (26.2)	4.16 (2.1)	− .49	.13	− 3.84	− .10	** < .001**	.04	.13	0.31	.01	.757
4th year	790 (19.4)	4.46 (2.18)	− .19	.132	− 1.44	− .03	.151	.082	.13	0.65	.02	.515
*Relationship status*												
Single	3150 (77.3)	4.35 (2.14)				†						†
In a relationship	703 (17.2)	4.43 (2.11)	.08	.09	0.88	.01	.381	< − .01	.08	− 0.02	< − .01	.983
Married	223 (5.5)	5.15 (2.21)	.80	.15	5.39	.08	** < .001**	.60	.14	4.36	.06	** < .001**
*Subject area*												
Science	2799 (68.7)	4.39 (2.14)				†						†
Arts	874 (21.4)	4.5 (2.15)	.11	.08	1.34	.02	.180	.11	.08	1.51	.02	.130
Commerce	403 (9.9)	4.33 (2.23)	− .06	.11	− .48	− .01	.632	.08	.10	0.75	.01	.451
*Socioeconomic status (SES)*												
Lower SES	437 (10.7)	4.56 (2.11)				†						†
Middle SES	1601 (39.3)	4.29 (2.15)	− .26	.12	− 2.28	− .06	**.023**	− .22	.11	− 2.10	− .05	**.036**
Upper SES	2038 (50.0)	4.46 (2.15)	− .09	.11	− .82	− .02	.412	− .11	.11	− 1.08	− .03	.282
*Residence*												
Urban	3209 (78.7)	4.43 (2.19)				†						†
Rural	867 (21.3)	4.31 (1.98)	− .12	.08	− 1.48	− .02	.140	− .14	.08	− 1.88	− .03	.061
*BMI*												
Normal	2646 (64.9)	4.47 (2.12)				†						
Underweight	505 (12.4)	3.96 (2.19)	− .51	.104	− 4.91	− .08	** < .001**	− .38	.10	− 4.01	− .06	** < .001**
Overweight	614 (15.1)	4.53 (2.21)	.064	.096	0.67	.01	.505	− .07	.09	− 0.84	− .01	.401
Obese	311 (7.6)	4.35 (2.14)	− .121	.128	− 0.95	− .02	.344	− .22	.12	− 1.92	− .03	.056
*Fitness goal*												
No	1132 (27.8)	3.46 (2.03)				†						†
Yes	2944 (72.2)	4.77 (2.08)	1.30	.07	18.04	.27	** < .001**	.84	.07	12.06	.18	** < .001**
*Perceived weight compared to before COVID*− *19*												
Gain	1750 (42.9)	4.5 (2.17)				†						
Loss	816 (20.0)	4.86 (2.19)	.36	.09	4.02	.07	** < .001**	.346	.082	4.23	.06	** < .001**
Same	1267 (31.1)	4.1 (2.07)	− .40	.08	− 5.09	− .09	** < .001**	− .157	.072	− 2.17	− .03	**.030**
Don't know	243 (6.0)	3.79 (1.91)	− .71	.15	− 4.85	− .08	** < .001**	− .223	.133	− 1.68	− .02	.093
*Guilty feelings about violating food rules*												
Always	1563 (38.3)	4.23 (1.91)				†						†
Sometimes	1547 (38.0)	5.32 (2.07)	1.09	.07	15.21	.24	** < .001**	.92	.07	13.25	.21	** < .001**
No	966 (23.7)	3.22 (1.97)	− 1.01	.08	− 12.44	− .20	** < .001**	− .80	.08	− 10.01	− .16	** < .001**
*Perceived problem*− *solving skills*												
Good	1401 (34.4)	4.76 (2.19)										
Moderate	2433 (59.7)	4.28 (2.09)	− .48	.07	− 6.72	− .11	** < .001**	− .347	.066	− 5.24	− .08	** < .001**
Poor	242 (5.9)	3.68 (2.09)	− 1.07	.15	− 7.25	− .12	** < .001**	− .646	.137	− 4.70	− .07	** < .001**
*Perceived social interaction*												
Good	1855 (45.5)	4.6 (2.16)				†						
Moderate	1760 (43.2)	4.37 (2.15)	− .23	.07	− 3.20	− .05	**.001**	− .109	.065	− 1.66	− .03	.096
Poor	461 (11.3)	3.77 (1.96)	− .82	.11	− 7.41	− .12	** < .001**	− .51	.103	− 4.98	− .08	** < .001**
*Smoking habits*												
Active smoker	447 (11.0)	4.19 (2.22)				†						†
Former smoker	171 (4.2)	4.67 (2.24)	.49	.19	2.52	.05	**.012**	.37	.17	2.15	.03	**.031**
Non smoker	3458 (84.8)	4.42 (2.13)	.24	.11	2.18	.04	**.029**	.08	.11	0.74	.01	.461

Bold values dictate that those are statistically significant

SD, Standard deviation; B, unstandardized regression coefficient; SE, standard error; β, standardized regression coefficient

^†^Reference category

**Table 2:** Distribution of all dietary items and association with orthorexia and food addiction

Food groups	Overall	Orthorexia			Food addiction		
	n (%)	Mean (SD)	t	*p*-value	Mean (SD)	t	*p*-value
*Starchy staples*^a^							
No	81 (2.0)	5.14 (2.11)	9.57	**0.002**	1.96 (1.45)	0.48	0.488
Yes	3995 (98.0)	4.39 (2.15)			1.85 (1.49)		
*Dark green leafy vegetables*							
No	1714 (42.1)	4.14 (2.03)	45.19	** < 0.001**	1.80 (1.50)	2.90	0.089
Yes	2362 (57.9)	4.60 (2.21)			1.88 (1.48)		
*Other vitamin A rich fruits and vegetables*^b^							
No	2130 (52.3)	4.15 (2.02)	62.72	** < 0.001**	1.85 (1.52)	0.04	0.847
Yes	1946 (47.7)	4.68 (2.24)			1.85 (1.46)		
Other fruits and vegetables^c^							
No	379 (9.3)	4.58 (2.16)	2.77	0.096	2.00 (1.54)	4.13	**0.042**
Yes	3697 (90.7)	4.39 (2.15)			1.83 (1.48)		
*Organ meat*							
No	3148 (77.2)	4.23 (2.03)	89.95	** < 0.001**	1.8 (1.47)	15.33	** < 0.001**
Yes	928 (22.8)	4.99 (2.43)			2.02 (1.53)		
*Meat and fish*^d^							
No	420 (10.3)	4.69 (2.07)	8.38	**0.004**	1.88 (1.48)	0.15	0.697
Yes	3656 (89.7)	4.37 (2.15)			1.85 (1.49)		
*Eggs*							
No	1221 (30)	4.24 (1.98)	10.11	**0.001**	1.78 (1.44)	4.28	**0.039**
Yes	2855 (70)	4.48 (2.21)			1.88 (1.51)		
*Legumes, nuts and seeds*							
No	1912 (46.9)	4.21 (1.99)	29.10	** < 0.001**	1.87 (1.53)	0.79	0.376
Yes	2164 (53.1)	4.58 (2.27)			1.83 (1.46)		
*Milk and milk products*							
No	1380 (33.9)	4.21 (2.05)	17.39	** < 0.001**	1.86 (1.56)	0.13	0.724
Yes	2696 (66.1)	4.51 (2.19)			1.84 (1.45)		

Bold values dictate that those are statistically significant

^a^The starchy staples food group is a combination of Cereals and White roots and tubers

^b^The other vitamin A rich fruit and vegetable group is a combination of vitamin A rich vegetables and tubers and vitamin A rich fruit

^c^The other fruit and vegetable group is a combination of other fruit and other vegetables

^d^The meat group is a combination of meat and fish

### Food addiction

FA were observed in 7.5% of participants. The mean mYFAS score was 1.85 (SD = 1.49) out of 9. All studied variables (*p* < 0.25), except for age and residence were selected as candidates of multivariable regression analyses (Table 3). In multivariable regression analyses, FA was associated with being female, actively smoking, being in their 1^st^ year of university, being married, having overweight or obesity, having fitness goals, having feelings of guilt always when violating food rules, poor social interactions, and weight loss as compared to the pre-COVID-19 period (Table 3). Participants who score highly on a measure of FA consumed more organ meats (t = 15.33, *p* < 0.001) and eggs (t = 4.28, *p* = 0.039) (Table 2).

**Table 3:** Bivariate and multivariable regression analyses by food addiction

Variables	Food addiction										
	Mean (SD)	Unadjusted estimates					Adjusted estimates				
		B	SE	t	ꞵ	*p*-value	B	SE	t	ꞵ	*p*-value
*Age*											
18–20 years	1.89 (1.48)				†						
21–23 years	1.83 (1.49)	− .06	.06	− .98	− .02	.329	–	–	–	–	–
24–28 years	1.88 (1.49)	− .01	.08	− .15	< − .01	.877	–	–	–	–	–
*Sex*											
Male	1.80 (1.44)				†					†	
Female	1.89 (1.53)	.08	.05	1.75	.03	.080	.15	.05	2.81	.05	**.005**
*Academic year of study*											
5th year/ Master	1.84 (1.4)				†					†	
1st year	2.00 (1.54)	.16	.09	1.72	.04	.086	.23	.10	2.44	.06	**.015**
2nd year	1.82 (1.47)	− .02	.09	− 0.26	− .01	.797	.02	.09	0.24	.01	.810
3rd year	1.78 (1.47)	− .06	.09	− 0.74	− .02	.462	− .01	.09	− 0.13	< − .01	.895
4th year	1.86 (1.53)	.01	.09	0.14	< .01	.89	.03	.09	0.34	.01	.731
*Marital status*											
Single	1.81 (1.48)				†					†	
In a relationship	1.95 (1.47)	.14	.06	2.20	.03	**.028**	.12	.06	1.92	.03	.055
Married	2.11 (1.62)	.30	.10	2.89	.04	**.004**	.26	.11	2.43	.04	**.015**
*Subject area*											
Science	1.83 (1.49)				†					†	
Arts	1.88 (1.47)	.06	.06	0.98	.02	.329	.04	.06	0.72	.01	.474
Commerce	1.92 (1.52)	.09	.08	1.17	.02	.244	.10	.08	1.31	.02	.192
*Socioeconomic status (SES)*											
Lower SES	1.75 (1.36)				†					†	
Middle SES	1.87 (1.54)	.12	.08	1.55	.04	.122	.12	.08	1.54	.04	.124
Upper SES	1.85 (1.47)	.11	.08	1.33	.04	.183	.09	.08	1.08	.03	.282
*Residence*											
Rural	1.83 (1.44)				†						
Urban	1.85 (1.5)	.02	.06	0.42	.01	.673	–	–	–	–	–
*BMI*											
Normal	1.77 (1.42)				†					†	
Underweight	1.84 (1.47)	.07	.07	0.98	.02	.327	.12	.07	1.65	.03	.098
Overweight	2.07 (1.65)	.30	.07	4.47	.07	** < .001**	.27	.07	4.06	.06	** < .001**
Obese	2.07 (1.68)	.30	.09	3.38	.05	**.001**	.26	.09	2.92	.05	**.003**
*Fitness goal*											
No	1.68 (1.42)				†					†	
Yes	1.91 (1.51)	.23	.05	4.41	.07	** < .001**	.14	.05	2.55	.04	**.011**
*Perceived weight compared to before COVID-19*											
Loss	1.89 (1.47)				†					†	
Gain	1.99 (1.54)	.10	.06	1.62	.03	.106	.11	.06	1.70	.04	.089
Same	1.63 (1.39)	− .26	.07	− 3.94	− .08	** < .001**	− .20	.07	− 2.94	− .06	**.003**
Don't know	1.83 (1.55)	− .06	.11	− 0.59	− .01	.556	− .01	.11	− 0.12	< − .01	.906
*Guilty feelings about violating food rules*											
Always	2.03 (1.56)				†					†	
Sometimes	1.8 (1.45)	− .40	.06	− 6.57	− .07	** < .001**	− .20	.05	− 3.69	− .06	** < .001**
No	1.63 (1.39)	− .23	.05	− 4.31	− .11	** < .001**	− .33	.06	− 5.26	− .09	** < .001**
*Social interaction*											
Poor	2.07 (1.63)				†					†	
Good	1.86 (1.47)	− .21	.08	− 2.70	− .01	**.007**	− .22	.08	− 2.92	− .08	**.004**
Moderate	1.78 (1.46)	− .29	.08	− 3.73	− .10	** < .001**	− .27	.08	− 3.51	− .09	** < .001**
*Smoking habits*											
Non smoker	1.83 (1.49)				†					†	
Active smoker	1.98 (1.5)	.15	.08	2.04	.03	**.042**	.25	.08	3.11	.05	**.002**
Former smoker	1.9 (1.42)	.07	.12	0.61	.01	.543	.14	.12	1.20	.02	.230

Bold values dictate that those are statistically significant

SD, Standard deviation; B, unstandardized regression coefficient; SE, standard error; β, standardized regression coefficient

^†^Reference category

### Dietary diversity

The mean score of the Dietary Diversity Scale (DDS) was 5.96 (SD = 1.56) out of 9. In bivariate regression analyses, all studied variables (*p* < 0.25) were selected as candidates of multivariable regression analyses (Table 4). According to multivariable regression analyses, DD was associated with older age (24–28 years), being of middle or upper SES, and having fitness goals (Table 4).

**Table 4:** Bivariate and multivariable regression analyses by dietary diversity

Variables	Dietary diversity										
	Mean (SD)	Unadjusted estimates					Adjusted estimates				
		B	SE	t	ꞵ	*p*-value	B	SE	t	ꞵ	*p*-value
*Age*											
24–28 years	6.06 (1.55)				†					†	
18–20 years	6 (1.62)	− .07	.08	− 0.82	− .02	.413	< − .01	.11	− .04	< − .01	.970
21–23 years	5.92 (1.54)	− .15	.06	− 2.30	− .05	**.022**	− .16	.08	− 1.99	− .05	**.047**
*Sex*											
Male	5.91 (1.60)				†					†	
Female	6 (1.52)	.09	.05	1.84	.03	.066	.03	.06	.57	.01	.566
*Academic year of study*											
1st year	5.89 (1.65)				†					†	
2nd year	5.98 (1.54)	.09	.06	1.15	.02	.250	.12	.08	1.43	.03	.154
3rd year	5.95 (1.51)	.06	.08	0.78	.01	.434	.12	.09	1.30	.03	.195
4th year	6 (1.60)	.11	.08	1.32	.03	.188	.08	.10	.80	.02	.423
5th year/Master	5.99 (1.50)	.10	.10	0.97	.02	.331	− .03	.13	− .23	− .01	.821
*Marital status*											
Single	5.95 (1.55)				†					†	
In a relationship	5.96 (1.56)	.01	.07	0.14	< .01	.891	− .01	.07	− .15	< − .01	.883
Married	6.18 (1.59)	.24	.11	2.20	.02	**.028**	.16	.11	1.45	.02	.148
*Subject area*											
Arts	5.85 (1.54)				†					†	
Science	6 (1.57)	.15	.06	2.40	.04	**.016**	.09	.06	1.48	.03	.140
Commerce	5.95 (1.50)	.10	.09	1.05	.02	.296	.06	.09	.64	.01	.524
*Socioeconomic status (SES)*											
Lower SES	5.68 (1.78)				†					†	
Middle SES	5.87 (1.56)	.19	.08	2.25	.06	**.025**	.18	.09	2.12	.06	**.034**
Upper SES	6.09 (1.49)	.41	.08	4.97	.13	** < .001**	.38	.09	4.49	.12	** < .001**
*Residence*											
Rural	5.82 (1.59)				†					†	
Urban	6 (1.55)	.19	.06	3.11	.05	**.002**	.09	.06	1.46	.02	.144
*BMI*											
Normal	5.94 (1.52)				†					†	
Underweight	5.9 (1.69)	− .039	.076	− 0.51	− .01	.608	− .01	.08	− .15	< − .01	.879
Overweight	6.06 (1.54)	.118	.07	1.70	.03	.093	.09	.07	1.29	.02	.198
Obese	6.03 (1.67)	.087	.093	0.93	.01	.351	.06	.09	.67	.01	.501
*Fitness goal*											
No	5.81 (1.54)				†					†	
Yes	6.02 (1.56)	.22	.05	3.97	.06	** < .001**	.21	.06	3.80	.06	** < .001**
*Perceived weight compared to before COVID-19*											
Same	5.97 (1.55)				†					†	
Gain	5.99 (1.52)	.016	.057	0.28	.01	.778	− .02	.06	− .33	− .01	.739
Loss	5.97 (1.61)	− .005	.07	− 0.08	< − .01	.938	− .04	.07	− .51	− .01	.608
Don't know	5.69 (1.63)	− .281	.109	− 2.58	− .04	**.010**	− .24	.11	− 2.25	− .04	**.025**
*Social interaction*											
Poor	5.81 (1.53)				†					†	
Good	6.05 (1.59)	.24	.08	2.95	.08	**.003**	.21	.08	2.56	.07	.011
Moderate	5.91 (1.52)	.10	.08	1.18	.03	.238	.06	.08	.77	.02	.441
*Smoking habits*											
Active smoker	5.86 (1.58)				†					†	
Former smoker	5.82 (1.53)	− .04	.14	− 0.29	− .01	.773	− .04	.14	− .30	− .01	.765
Non smoker	5.98 (1.56)	.12	.08	1.57	.03	.117	.12	.09	1.42	.03	.155

Bold values dictate that those are statistically significant

SD, Standard deviation; B, unstandardized regression coefficient; SE, standard error; β, standardized regression coefficient

^†^Reference category

## Discussion

It is important to identify individuals who score highly on measures of FA and ON to intervene and manage EDs among younger adults in a low-resource country like Bangladesh [34]. We believe the current study is the first to report on ON, FA and DD among Bangladeshi students during the COVID-19 pandemic period. We found that 7.5% and 1.7% of the participants had FA and ON, respectively, with 43.8% Health Fanatic eating attitude. Our findings also indicated that older age, being married, formerly smoking, and having fitness goal and guilty feelings about violating food rules were associated with ON. FA was associated with being female, active smoking, married, overweight and obese. Students with ON consumed more legumes, nuts, seeds and vegetables, including dark green, leafy vegetables, whereas students with FA consumed more organ meats and eggs. Furthermore, being younger aged, belonging to lower SES, living in rural regions, and not having fitness goals were associated with lower DD.

In the present study, the mean BOT score was 4.41 ± 2.15 [close to the “health fanatic” range] which is comparable to a previous study (mean score = 4.71) among college students using the BOT [59]. Moreover, the study shows 43.8% of the participants may have a risk of developing ON as they exhibited Health Fanatic eating attitude [18]. The prevalence of ON is higher in the present study compared to a previous study among dietetics students [21]. This discrepancy might be attributed due to the different samples, socio-demographic statuses, or instruments used, among other factors. This study showed that among individuals with ON, most were from science backgrounds (67.6%), as compared to the commerce (9.9%) and arts (22.5%) students. Students from science background have health-related majors in their academic subjects that offer information on proper diet and health [69]. Their frequent exposure to food and nutrition information has been identified as a factor in the development of EDs [70]. In the present study, ON was more prevalent among 1st and 2nd year students compared to Master’s students. Starting at a university can be a time of great change, where students enter unfamiliar environments, engage with new people, and consider their futures and aspirations; they may also experience increased pressures, academically and socially [40]. It is possible that this new independence and its associated pressures may influence interests in food, including restrictions [71]. Being married, as compared to being single, was associated with ON, consistent with findings from a previous study in India [72]. A Turkish study [22] had found that being overweight or obese was associated with ON; however, this study did not find such an association. Rather, underweight individuals were less likely to have ON compared to respondents who had normal BMIs.

Our data suggest that students who had fitness goals scored higher on the BOT than students who did not. Body image, attitude, perfectionist personality features and prior histories EDs have been linked to ON [73], which may in part explain relationships between ON and fitness goals. Individuals with ON may obsessively follow eating rules, as consuming only ‘good’ or ‘correct’ foods may be relaxing, stress-lowering experiences for them [10, 74]. Social interactions have been suggested to be poor among those with ON [74], perhaps as stringent food rules do not allow for social flexibility or may create barriers to friendships, but this study found the opposite. The COVID-19 pandemic has significantly reduced face-to-face social interactions; rather, people are now often interacting with each other online through different social media. As a result, the new normal life may permit good social interactions without in-person meetings that may involve dining. Such online meetings may facilitate good social interactions in individuals with ON without challenging their orthorexic eating patterns. This possible explanation is currently speculative and requires further examination.

This study also found that ON is higher among non-smoking and formerly smoking individuals, perhaps because people with ON never smoked or quit smoking in order to lead more healthy lives. Moreover, it is also possible that individuals who quit smoking or did not smoke were leading healthy lives which increased potential risk for ON. These and other possible explanations warrant future exploration. A prior study linked ON to smoking when smoking motivations were for weight control [75], and this possibility warrants further exploration.

This study also found that students who reported having lost weight during the COVID-19 pandemic compared to the pre-COVID-19 period were more likely score high on ON measure. The omission or restriction of certain food groups or products could explain this weight loss among individuals with ON [9]. Further assessment during and beyond the pandemic could provide further insights into such relationships.

The current study estimated a lower prevalence of FA in Bangladeshi university students than that (10.3%) previously reported among students in the United States [34]. Notably, the reported prevalence of our study is somewhat parallel with that of a nationally representative study of German adults, which estimated prevalence of FA at 7.9% [76]. Higher consumption of fast food in Bangladeshi younger people, particularly university students may in part account for food addiction [35] as highly processed fast food has been proposed to have a higher addictive potential [77]. In the present study, older, female and 1^st^-year students were more likely to have FA, in line with prior studies [31, 34, 78]. The current study found that married students had higher FA than the unmarried students. This finding differs from a prior study indicating that single people have FA scores than married people [32]. We observed a strong relationship between FA and BMI in the multivariable regression analysis, suggesting that people with higher BMIs (overweight/obese) were more likely to exhibit features of FA, consistent with previous findings [31, 34, 64]. FA is associated with compulsive consumption of calorie-dense foods, which increases the likelihood of obesity [79].

The current study found associations between FA and active smoking, consistent with prior research [33]. Highly palatable foods have been proposed to promote food cravings by activating brain reward pathways, and it is speculated that smoking may be linked to FA by similar neural mechanisms [33, 80]. The current study also linked fitness goals to FA. Individuals with higher score a measure of FA are more likely to be obese [31]; thus, they may have increased concern regarding body appearances and have fitness goals to lose weight. In the present study, FA was associated with feeling guilty about violating food rules. It is plausible that people who violate food rules more frequently are more likely to have higher score on FA measure, and this possible explanation should be further examined in future studies [81].

The food groups with the highest likelihoods of consumption in the 24 h period preceding the survey included starchy staples (98%), followed by vitamin-A-rich fruits and vegetables (90.7%), and animal proteins (89.7%). This food-consumption pattern is similar to a previous study among rural children in Bangladesh [82]. Students with higher score on ON measure consume more legumes, nuts, seeds, and vegetables than the students who didn’t have ON. Students with higher score on FA measure consumed more organ meats and eggs than their counterparts. Resonating with these findings, tertile distributions from a previous study show that many students with ON were in upper tertile of “legumes and nuts” (43.9%) and “fruits & vegetables”(42.9%) [83]. Vegetarian diets have been linked to ON [84], whereas FA has been linked to consumption of meat [85] and eggs [86], consistent with our findings. Prior data suggest that FA diets are richer in calories from fats and proteins [31], cholesterol, saturated fats, and animal proteins [87].

The study found that older students had higher DD which is inconsistent with a previous study from Algeria [88]. In Bangladesh, most younger students tend to reside in university settings (e.g., dormitories) rather than with their families, which may be a possible reason for lower DD among younger students in the present study. The current study found that students who had fitness goals and better social interactions consumed more diversified foods. This finding warrants further study to understand the rationale behind this finding as no previous studies have investigated associations between DD and fitness goals and social interactions. Participants from higher socio-economic status groups (monthly income greater than 30 thousand BDT [> 352 USD]) exhibited higher DD, which is in line with a previous study from Bangladesh during the COVID-19 pandemic [89]. Amounts of money spent on food have been positively correlated with DD [90], and people from higher SES groups are more likely to spend more money on food. A prior study during the COVID-19 pandemic showed that precariously employed families (households with day laborers, private jobholders, businessmen, and farmer family heads) reported lower DD [41]. Rural participants had lower DD compared to urban participants, which is supported by previous findings [41]. Urban people are more likely to order food online, which may be a possible reason for the higher DD in urban areas [91]. This finding implies the importance of tracking diet-related policies and practices (e.g., access to a variety of foods) in lower SES groups and rural areas, particularly during times when countries are encountering additional strains (e.g., financial hardships, disrupted food production, and impaired distribution systems) related to the COVID-19 pandemic.

### Strengths and limitations

To the best of our knowledge, this is the first study to estimate the prevalence of and explore factors associated with ON among Bangladeshi university students, and is among a limited number of such studies worldwide. A strength of our study includes the large number of students from across Bangladesh. Finally, pre-testing the survey prior to data collection strengthened the instrument, improving its appropriateness for the setting and enhancing clarity for participants.

The study has some limitations to consider, especially when extrapolating the findings. The online cross-sectional survey design limited our ability to ascribe causality to any associated factors. A longitudinal study design would be better suited to understand FA and ON in this regard. While using an anonymous online survey may improve the accuracy of disclosure among respondents for sensitive inquiries related to eating behaviors [92], it is possible that aspects may be subject to reporting biases. Although many self-reported measures (e.g., height and weight) are showed as generally reliable in previous study [93], it might be a reason of biases. Also, the questions about social interaction and problem-solving skills had few response alternatives which should be documented as another limitation. Furthermore, the convenience sampling approach and use of self-reported data may limit the generalizability of the findings by introducing recall, selection, and social desirability bias. The common method bias may cause inflate relationships between study variables. Lastly, the prevalence rate of ON and FA should be interpreted with caution because of different cut-off points usage in different studies. Despite these limitations, the findings from this study suggest the need for conducting further studies on FA, ON and DD to assist public health authorities and policymakers in implementing appropriate interventions.

### Public health recommendations

Disordered eating behaviors, including ON and FA, among students require increased attention. Identifying those who are at higher risk may help guide appropriate public policy initiatives such as, speculatively, zoning requirements for fast food restaurants in Bangladesh. Furthermore, frequent screening and awareness programs may help lower risks of disordered eating and their impacts on health and overall well-being among university students. Students belonging to lower socio-economic status groups and having rural residences appeared to have lower DD. Food and nutritional support may be an important aspect of social protection programs to help guarantee that the most disadvantaged populations have access to diversified foods by safeguarding their buying power and distributing food directly, if necessary. Nutritional awareness programs focusing on DD and healthy eating habits should be implemented for students.

## Conclusions

This study investigated factors linked to ON, FA and lower DD among Bangladeshi university students. The gathered information may help facilitate early identification and intervention to prevent negative health consequences arising from these factors. Participants who had fitness goals and guilty feelings about violating food rules and who were older aged, married and formerly smoked were more likely to report ON, whereas being female, married, or a 1^st^-year student, active smoking, and being overweight or obese were associated with FA. These findings challenge the notion that disordered eating is an exclusively “Western” problem, and emphasize the importance of investigating ON and FA across cultures. Future investigations with wider and more diverse cohorts could provide further insights into ON, FA and DD in order to promote the public health across jurisdictions.

## Data Availability

The datasets generated during and/or analyzed during the current study are not publicly available due to patient confidentiality.
